# Optimization of Ultrafast Proteomics Using an LC-Quadrupole-Orbitrap Mass Spectrometer with Data-Independent Acquisition

**DOI:** 10.1021/acs.jproteome.2c00121

**Published:** 2022-08-01

**Authors:** Masaki Ishikawa, Ryo Konno, Daisuke Nakajima, Mari Gotoh, Keiko Fukasawa, Hironori Sato, Ren Nakamura, Osamu Ohara, Yusuke Kawashima

**Affiliations:** † Laboratory of Clinical Omics Research, Department of Applied Genomics, Kazusa DNA Research Institute, Kisarazu, Chiba 292-0818, Japan; ‡ Institute for Human Life Innovatiaon, Ochanomizu University, Bunkyo-ku, Tokyo 112-8610, Japan

**Keywords:** Q-Orbitrap MS, data-independent acquisition, HT proteomics, Short LC gradients

## Abstract

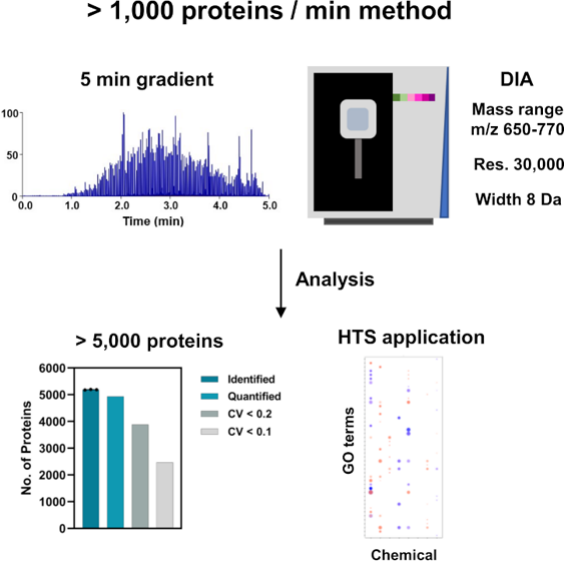

Proteomics has become an increasingly important tool in medical and medicinal applications. It is necessary to improve the analytical throughput for these applications, particularly in large-scale drug screening to enable measurement of a large number of samples. In this study, we aimed to establish an ultrafast proteomic method based on 5-min gradient LC and quadrupole-Orbitrap mass spectrometer (Q-Orbitrap MS). We precisely optimized data-independent acquisition (DIA) parameters for 5-min gradient LC and reached a depth of >5000 and 4200 proteins from 1000 and 31.25 ng of HEK293T cell digest in a single-shot run, respectively. The throughput of our method enabled the measurement of approximately 80 samples/day, including sample loading, column equilibration, and wash running time. We demonstrated that our method is applicable for the screening of chemical responsivity via a cell stimulation assay. These data show that our method enables the capture of biological alterations in proteomic profiles with high sensitivity, suggesting the possibility of large-scale screening of chemical responsivity.

## Introduction

In recent years, mass spectrometry (MS)-based proteomics have been applied in both clinical and medicinal fields, as represented by biomarker discovery and drug development.^1−4^ An improvement in the analytical throughput is a requisite for these applications because of the requirement of analyzing a large number of samples. Usually, nano liquid chromatography (LC) is used in MS-based proteomics because of its high sensitivity and low flow rate that improves the ionization efficiency of peptides by electrospraying. Recent in-depth proteomics, comprising nano LC and data-independent acquisition (DIA/SWATH), have reached a depth of more than 7,000 protein identifications from mammalian tissues and cells in a single-shot run.^5−8^ However, in-depth proteomics require a longer acquisition time, making it challenging to improve the analytical throughput. Therefore, new chromatographic and mass spectrometric approaches are required to overcome this challenge. The sensitivity of MS has improved over several years, making micro-flow LC possible for high-throughput (HT) proteomic analysis. Recently, high-flow-rate LC-based methods have been developed.^9−14^ Bache et al. reported the identification of 5,446 proteins from HeLa cells using a 20 min-per-sample method based on a novel LC system combined with online solid phase extraction and micro-/nano-flow LC.^9^ Further improvements in the analytical throughput have been attempted by combining the applied-DIA/SWATH with quadrupole time-of-flight MS (Q-TOF MS)^12,13^ along with ion mobility spectrometry.^12^ Meier et al. reported approximately 3000 proteins from HeLa cells using 5.6-min gradient LC and ion mobility/applied-SWATH.^12^ Messner et al. obtained 5004 proteins from K562 cells using 5-min micro-flow LC and applied SWATH.^13^ Consequently, “ultrafast proteomics” have become feasible for large-sample studies.

Q-Orbitrap and Q-TOF MS have been widely used in the proteome research because of their high resolution and sensitivity, especially in DIA.^15,16^ DIA utilizes isolation windows to co-isolate and elute fragment peptides regardless of their signal intensity, thereby providing a systematic collection of peptide fragments. Consequently, DIA allows the identification of peptides with high sensitivity and improved reproducibility. In DIA, a narrow isolation window width with higher mass resolution (required longer transient time) decreases the spectral complexity.^5^ Thus, Q-Orbitrap MS, which enables the regulation of mass resolution via the injection of precursor/product ions in the C-trap into the Orbitrap mass analyzer, has a potential advantage in improving sensitivity; ions in Q-Orbitrap MS are initially accumulated and/or fragmented in the ion routing pole and then transferred to the C-trap.^14^ Q-Orbitrap is considered unsuitable for HT proteome analysis because of its relatively slow scan speed arising from ion accumulation; therefore, Q-TOF MS has been mainly applied for the development of HT proteomic methods.^11−13^ Meanwhile, it has not been fully investigated whether Q-Orbitrap MS is unsuitable for HT proteomics. The mass range, mass resolution, and isolation window width in Q-Orbitrap MS for HT proteomic analysis remain poorly understood.

Herein, we evaluated DIA parameters combined with 5-min gradient LC and Q-Orbitrap MS. Based on the findings, we established a simple method with high throughput and deep proteome coverage without any specialized equipment. Moreover, for future high-throughput proteomic applications, we performed cell stimulation assays using chemical substances and continuous proteome analysis of stimulated cell samples.

## Experimental Section

### Cell Culture

HEK293T cells (Lot No. 19I006; ECACC, Wiltshire, U.K.) were cultured in a 15 cm dish at 80% confluence in Dulbecco’s modified Eagle’s medium (DMEM; Thermo Fisher Scientific, Waltham, MA) containing 10% fetal bovine serum (FBS; Thermo Fisher Scientific) and 1% penicillin/streptomycin (Fujifilm Wako, Osaka, Japan) at 37 °C in a 5% CO_2_ incubator. The cells were detached using TrypLE Express (Thermo Fisher Scientific) at 37 °C for 5 min and then collected in phosphate-buffered saline (PBS; Nacalai Tesque, Kyoto, Japan). After collection, 1 × 10^6^ cells were precipitated at 1000 rpm and 4 °C for 2 min. The precipitate was frozen immediately at −80 °C and kept frozen until protein extraction.

### Sample Preparation for LC-MS/MS

Cell samples were prepared as described in a previous study.^17^ In brief, precipitates were dissolved in 100 mM Tris-HCl (pH 8.5) containing 2% sodium dodecyl sulfate (SDS) using BIORUPTOR BR-II (SONIC BIO Co., Kanagawa, Japan) with settings at “High” and “30 s On/Off” cycle for a duration of 5 min. The extracted proteins were quantified using a Pierce BCA Protein Assay Kit (Thermo Fisher Scientific) at 1000 ng/μL. The extracts were reduced with 10 mM dithiothreitol for 30 min at 50 °C, followed by alkylation with 30 mM iodoacetamide for 30 min at 25 °C in the dark. Protein purification and digestion were performed using the sample preparation (SP3) method.^17,18^ The tryptic digestion was performed using 500 ng/μL Trypsin/Lys-C Mix (Promega, Madison, WI) overnight at 37 °C. Cell digests were purified using GL-Tip SDB (GL Sciences, Tokyo, Japan) according to the manufacturer’s protocol. The peptides were dissolved again in 3% acetonitrile (ACN) containing 0.1% trifluoroacetic acid (TFA) and then quantified using a Lunatic UV/Vis absorbance spectrometer (Unchained Labs, Pleasanton, CA).^19^

### 5-Min Gradient LC-MS/MS

Two mobile phases, A and B, containing H_2_O and 80% ACN/H_2_O (4/1, v/v) were prepared in 0.1% formic acid (FA). Then, 1 or 2 μL of the digested peptides were loaded directly using a 150 μm-inner-diameter × 5 cm capillary column (Aurora C18, particle size 1.6 μm, 120 Å; IonOpticks, VIC, Australia) at 8.0 μL/min and 40 °C for 4.0 min; to this end, an UltiMate 3500 RSLC nano system (Thermo Fisher Scientific) equipped with a 10 μL injection loop was employed. The linear gradient was executed at 1.5 μL/min and 40 °C, and the gradient conditions were as follows: 0–4.0 min, B 2% held; 4.0–4.1 min, B 2–5%; 4.1–8.0 min, B 5–36%; 8.0–8.8 min, B 36–95%; and 8.8–9.0 min, B 95% held. MS acquisitions were initiated 4.0 min after sample injection. The eluted peptides were detected using a quadrupole Orbitrap Exploris 480 hybrid mass spectrometer (Thermo Fisher Scientific) equipped with an ING Nano ion source (AMR Inc., Tokyo, Japan). The total acquisition time of one sample was 9.5 min, corresponding to the sum of the sample loading time, gradient time, and overhead time, which were 4.0, 5.0, and 0.5 min, respectively. The wash running time between the samples was 8.0 min. Overall, the injection time was 17.5 min (approximately 80 samples/day).

### Screening of Suitable DIA Parameters for 5-Min Gradient LC

To evaluate DIA with 5-min gradient LC, we designed 46 DIA methods comprising five groups with different combinations of the precursor mass range (narrow range, 120 Da; wide range, 240 Da), mass resolutions (7500, 15,000, 30,000), and isolation window width (4, 8, 16 Da). The five groups were marked as Res7.5k/W4, Res15k/W8, Res30k/W16, Res15k/W4, and Res30k/W8, respectively, in the present study (Table 1). In all methods, the MS1 scan range was set as full scan, *m*/*z* 400–1100, and mass resolution as 7500. The auto gain control (AGC) target was set as 1 × 10^6^ and the maximum injection time as 10 ms for MS1. The AGC target for MS2 was set as 3 × 10^6^, at 28% normalized collision energy. In DIA, the maximum injection time values at mass resolutions of 7500, 15,000, and 30,000 were set at 7, 22, and 54 ms, respectively. The overlapping windows of the Res7.5k/W4, Res15k/W8, Res30k/W16, Res15k/W4, and Res30k/W8 groups were set as 2, 4, 8, 2, and 4 Da, respectively.

**Table 1:** Information of 46 DIA Methods for 5-min Gradient LC

wide mass range method			narrow mass range method	
resolution/isolation window/mass range			resolution/isolation window/mass range	
7500/4 Da	15000/8 Da	30000/16 Da	15000/4 Da	30000/8 Da
Res7.5kW4/400-640	Res15k/W8/400-640	Res30k/W16/400-640	Res15k/W4/400-520	Res30k/W8/400-520
Res7.5k/W4/450-690	Res15k/W8/450-690	Res30k/W16/450-690	Res15k/W4/450-570	Res30k/W8/450-570
Res7.5k/W4/500-740	Res15k/W8/500-740	Res30k/W16/500-740	Res15k/W4/500-620	Res30k/W8/500-620
Res7.5k/W4/550-790	Res15k/W8/550-790	Res30k/W16/550-790	Res15k/W4/550-670	Res30k/W8/550-670
Res7.5k/W4/600-840	Res15k/W8/600-840	Res30k/W16/600-840	Res15k/W4/600-720	Res30k/W8/600-720
Res7.5k/W4/650-890	Res15k/W8/650-890	Res30k/W16/650-890	Res15k/W4/650-770	Res30k/W8/650-770
Res7.5k/W4/700-940	Res15k/W8/700-940	Res30k/W16/700-940	Res15k/W4/700-820	Res30k/W8/700-820
Res7.5k/W4/750-990	Res15k/W8/750-990	Res30k/W16/750-990	Res15k/W4/750-870	Res30k/W8/750-870
			Res15k/W4/800-920	Res30k/W8/800-920
			Res15k/W4/850-970	Res30k/W8/850-970
			Res15k/W4/900-1020	Res30k/W8/900-1020

### Cell Stimulation Assay

HEK293T cells were cultured in six-well plates (Thermo Fisher Scientific) at 70% confluence in DMEM, and then this medium was replaced with another medium containing chemical substances. All chemical substances were purchased from Avanti Polar Lipids (AL). The cells were stimulated with different concentrations (0.1, 1.0, and 10 μM) of 1-myristoyl-2-hydroxy-*sn*-glycero-3-phosphate (LPA(14:0)), d-erythro-sphingosine-1-phosphate (S1P), d-erythro-sphingosine 18:1 (Sph), 1,2-dioleoyl-*sn*-glycero-3-phospho-(1′-myo-inositol-3′-phosphate) (PI3P), 1,2-dioleoyl-*sn*-glycero-3-phospho-(1′-myo-inositol-4′-phosphate) (PI4P), 1,2-dioleoyl-*sn*-glycero-3-phospho-(1′-myo-inositol-5′-phosphate) (PI5P), d-myo-inositol-1,4,5-triphosphate (IP3), and d-myo-inositol-1,3,4,5-tetraphosphate (IP4). After 24 h of stimulation, individual cells were collected by lysis in 400 μL of 2% SDS in Tris-HCl (pH8.5) followed by a PBS wash. Proteomic samples were prepared using the above-mentioned protocols, and samples were placed in 96-well plates (Eppendorf, Hamburg, Germany). The cell stimulation assay was performed in triplicate.

### Data Processing

The raw data files were converted to mzML files (using parameters “peak picking” and “demultiplex”) by ProteoWizard (version: 3.0.19254). The converted mzML files were transformed into DIA files for search using DIA-NN^11,20^ (version:1.8, https://github.com/vdemichev/DiaNN). Peptides and proteins were identified and quantified from each DIA file using a two-step search. First, a spectral library for the library-free search was generated from the human protein sequence database (UniProt id UP000005640, reviewed, canonical, 20381 entries) using DIA-NN. The DIA-NN search parameters were as follows: protease, trypsin/P; missed cleavages, 1; peptide length range, 7–45; precursor charge range, 2–4; fragment ion *m*/*z* range, 200–1800; mass accuracy, 15 ppm; static modification, cysteine carbamidomethylation; and enabled “Remove likely interferences” and “Use isotopologues.” The precursor mass range was varied according to the DIA method (Table 1). Additional commands were set as follows: peak translation and -relaxed-prot-inf. The protein identification threshold was set at <1% for both peptide and protein false discovery rate (FDR) values. Next, a protein search was performed again using the generated specific spectral library. The protein quantity was measured as the sum of the quantities of the four best precursors. In addition, the raw data files were analyzed against a human spectral library using Scaffold DIA (Proteome Software Inc., Portland, OR). The human spectral library was built from the human protein database (UniProt id UP000005640, reviewed, canonical) established by Prosit.^21^ The Scaffold DIA search parameters were as follows: experimental data search enzyme, trypsin; maximum missed cleavage sites, 1; precursor mass tolerance, 15 ppm; fragment mass tolerance, 15 ppm; and static modification, cysteine carbamidomethylation. The peptide and protein FDR values were set to <1%. Peptide quantification was performed using the EncyclopeDIA^22^ algorithm in Scaffold DIA. For each peptide, four fragment ions of the highest quality were selected for quantification. Protein values were normalized using linear median normalization between samples.

### Statistical Analysis of the Cell Stimulation Assay

Statistical quantitative analysis was performed using Perseus.^23^ Samples were categorized into individual dose groups (0.1, 1.0, and 10 μM) for each substance. The intensity values were log2-transformed, and proteins that quantified more than 70% of each group were chosen. Normalization was performed by width adjustment prior to imputation of missing values (downshift = 1.8, width = 0.3). One-way ANOVA for each substance group was performed, and significantly different proteins (*p* < 0.05) were extracted. Subsequently, hierarchical clustering was performed using the extracted proteins, and characteristic clusters were selected for each group. All other statistical analyses were performed using GraphPad Prism 9 (GraphPad Software, San Diego, CA).

### Pathway and Gene Ontology (GO) Enrichment Analysis

Pathway analysis was performed by the enrichment analysis of WikiPathway using Enricher (https://maayanlab.cloud/Enrichr/).^24^ The proteins with a significant difference (*p* < 0.05) between each stimulation and control were analyzed by this platform. Gene ontology enrichment analysis was performed for each cluster using Metascape (https://metascape.org/gp/index.html#/main/step1). The top five terms of the adjusted *p*-value in the biological process were selected in each cluster, and the dot plot was generated using R software (ggplot2 library). To evaluate the Res30k/W8/650–770 method, we performed GO enrichment analysis using DAVID (https://david.ncifcrf.gov/home.jsp) and extracted the proteins with GO terms MF of “kinase activity,” “protein kinase activity,” “receptor signaling protein serine/threonine kinase activity,” “transcription coactivator activity,” “transcription factor binding”, and “GTPase activity.”

## Results and Discussion

### Optimization of DIA for 5-Min Gradient LC

**Figure 1:**
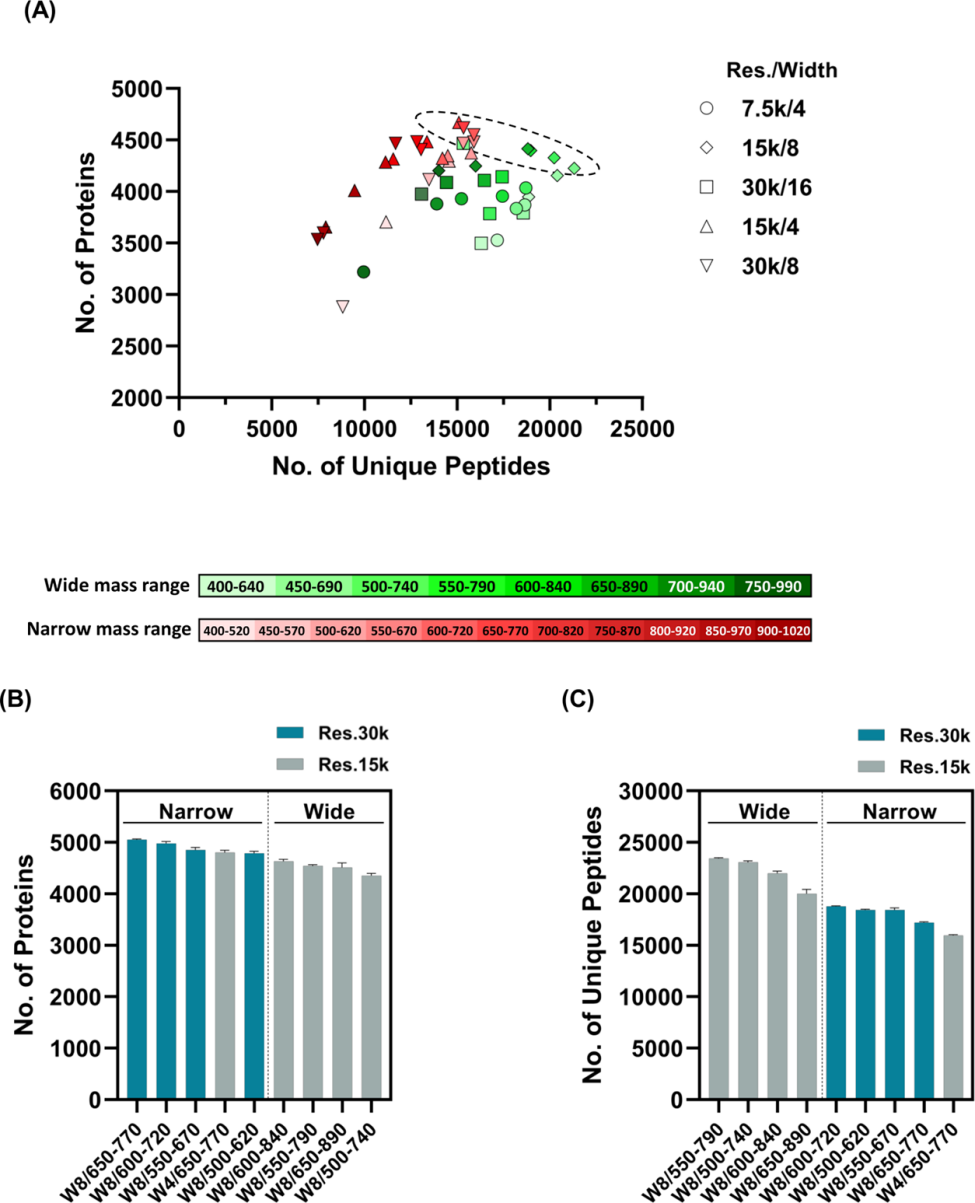
Screening of suitable DIA with 5-min gradient LC. (A) The graphs indicate the number of proteins and peptides from 46 DIA. The numbers of proteins and peptides are plotted in longitudinal and horizontal axes, respectively. Individual identifications were obtained from 46 methods in a single-shot run. The graphs indicate the number of identified proteins (B) and peptides (C) from selected nine DIA methods. Individual DIA was measured in triplicate. In the graph, “Narrow” and “Wide” show precursor mass range in DIA for each method (120 and 240 Da).

Q-TOF MS has been utilized in ultrafast proteomics because of its fast scan rate in high-resolution MS.^11−13^ This study aimed to establish an ultrafast method based on Q-Orbitrap MS by optimizing DIA with fast LC. We constructed 46 methods combining a different number of isolation windows, mass ranges, and resolutions, with the same scan cycle time, to evaluate DIA systematically for 5-min gradient LC (Table 1). We then screened the DIA with a higher number of identified proteins and peptides from the above 46 methods using 5-min gradient LC (Figure S1). Nine DIA methods (Res30k/W8/500–620, Res30k/W8/550–670, Res30k/W8/600–720, Res30k/W8/650–770, Res15k/W4/650–770, Res15k/W8/500–740, Res15k/W8/550–790, Res15k/W8/600–840, and Res15k/W8/650–890) showed high protein and peptide identification (Figure 1A, Dashed line). In the precursor mass range, these methods cover *m*/*z* 500–890 (starting to end at Res30k/W8d/500–620 to Res15k/W8/650–890; Table 1). The number of identified peptides in the MS range of *m*/*z* 400–640 and 450–690 exceeded 15,000, but few proteins were identified. In the low MS range, many peptides were identified because peaks of long peptides of triple charge or higher were observed in addition to short peptide peaks. However, owing to the increase in the number of peptide peaks detected, the MS2 pattern of DIA became more complicated, making it difficult to identify trace peptides. In higher MS ranges such as *m*/*z* 700–940, 750–990, 850–970, and 900–1020, fewer peptide peaks were observed, and the number of identified proteins did not increase. For shorter gradients, MS ranges of approximately *m*/*z* 500–890 were a suitable range that allowed us to increase the number of identified proteins, which was attributed to the combination of the number of peptides detected and the reduced complexity of MS2.

To verify the results of this screening, we measured the nine selected methods in triplicate and compared the proteins and peptides. Consequently, the methods with narrow precursor mass ranges (120 Da: Res15k/W4/650–770, Res30k/W8/650–770, Res30k/W8/600–720, Res30k/W8/550–670, and Res30k/W8/500–620) indicated a higher number of protein identifications than the wide mass range methods (240 Da: Res15k/W8/650–890, Res15k/W8/600–840, Res15k/W8/550–790, and Res15k/W8/500–740) (Figure 1B and Table S1). In contrast, there were fewer peptide identifications in the narrow mass range than in the wide mass range (Figure 1C and Table S1). Importantly, more peptides were co-eluted in the shorter LC gradient. A narrow mass range leads to a decrease in the number of detectable peptides, thus reducing spectral complexity. Thus, a narrow mass range is effective for improving protein identification in the shorter gradient LC.

Among the narrow methods, Res30k/W8/650–770 showed the highest protein identification, with an average of 5,055 proteins (Figure 1B and Table S1). Moreover, Res30k/W8/650–770 showed the highest reproducibility with respect to protein quantities (median coefficient of variation (CV) 11.6%, Table S1). To verify the protein identification of Res30k/W8/650–770 using a search algorithm other than DIA-NN, we reanalyzed the data obtained from the nine methods (indicated in Figure 1B,C) using Scaffold DIA with EncyclopeDIA. Results showed that the Res30k/W8/650–770 method showed the highest protein identifications even for Scaffold DIA software, although the protein and peptide identifications in Scaffold DIA were fewer than those in DIA-NN (Figure S2 and Table S2). In addition, protein and peptide identifications for the nine methods showed similar trends in Scaffold DIA (Figure S2 and Table S2); *i.e*., the number of protein and peptide identifications in narrow methods was higher and fewer, respectively, compared with those in wide methods. These results suggest that our method not only works with DIA-NN but also with other software. Based on these results, we determined that Res30k/W8/650–770 is the most efficient DIA for combining 5-min gradient LC and Q-Orbitrap MS, that is, precursor mass range, *m*/*z* 650–770; mass resolution, 30,000; and isolation window width, 8 Da. The throughput of our method enabled the measurement of approximately 80 samples/day, including the sample loading, column equilibration, and wash running time.

### Evaluation of Proteome Coverage and Reproducibility of Optimized DIA Parameters

**Figure 2:**
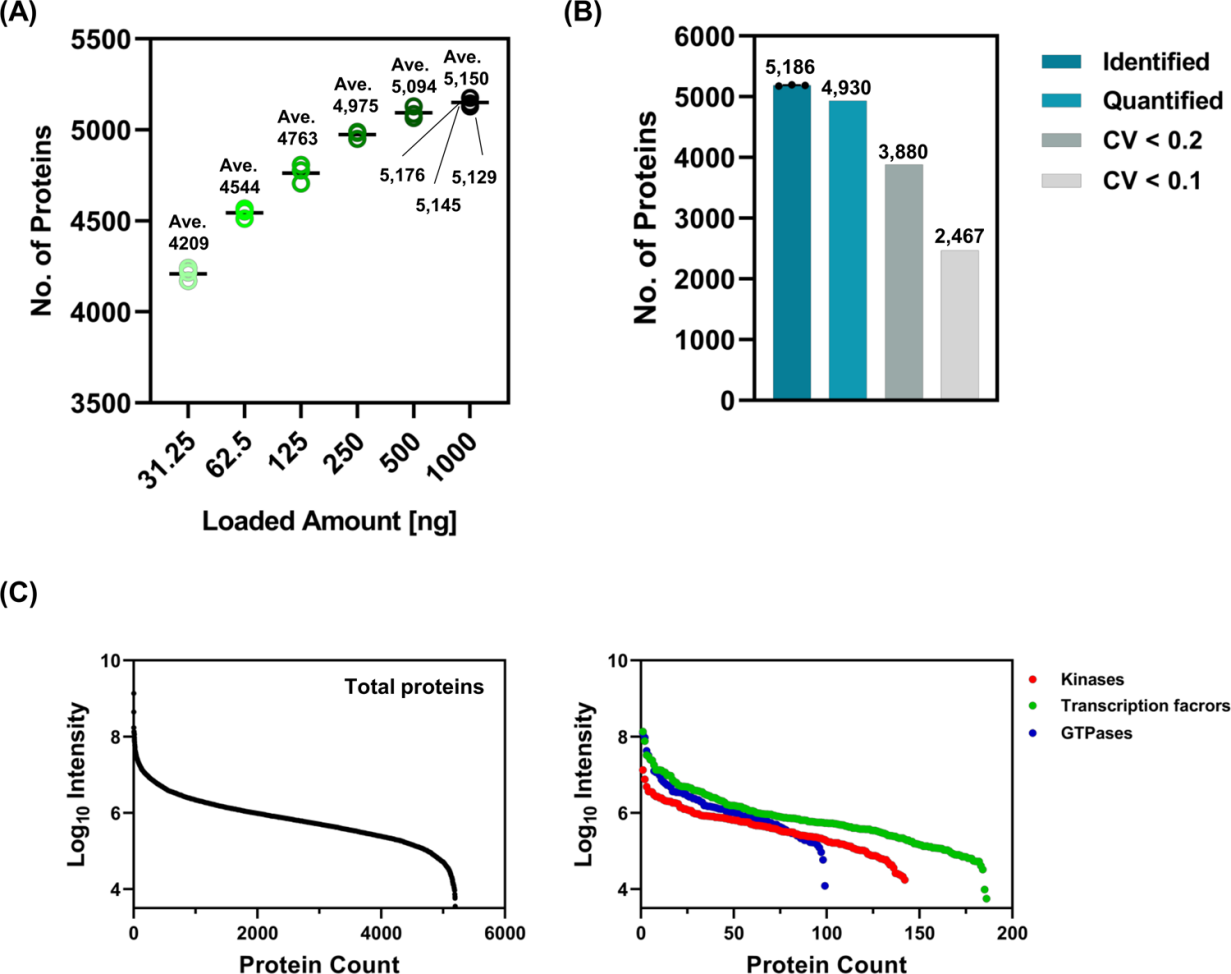
Proteome coverage and reproducibility of the Res30k/W8/650–770 method. (A) The plotted graphs indicate the number of protein identifications in a single-shot run when injected with 31.25, 62.5, 125, 250, 500, and 1000 ng of HEK293T cell digest. (B) The bar graphs indicate the number of proteins using a 1000 ng cell digest with triplicate runs. The average identifications are indicated in the bar graph identified above. (C) The graphs plot protein counts of all proteins (left panel), kinases, transcription factor-related proteins, and GTPases (right panel). Kinases were summarized using the protein-annotated GO terms, “kinase activity”, “protein kinase activity,” and “receptor signaling protein serine/threonine kinase activity.” Transcription factors were summarized using the protein-annotated GO terms “transcription coactivator activity” and “transcription factor binding.” Overlapping proteins in each term were unified before summarization.

To further examine the proteome coverage and robustness of the optimized DIA parameters, we measured 31.25, 62.5, 125, 250, 500, and 1000 ng of cell digests in a single-shot run. Our method showed the highest number of identified proteins for 1000 ng of digest injection, whereas the difference in the number of identified proteins was small between 500 and 1000 ng of digest injection (Figure 2A). The average numbers of identified proteins in the 31.25, 62.5, 125, 250, 500, and 1000 ng digests were 4209, 4544, 4763, 4975, 5094, and 5150, respectively. When measuring based on our method in a single-shot run in triplicate, more than 5000 proteins, namely, 5176, 5145, and 5129 proteins, were identified (Figure 2A).

**Figure 3:**
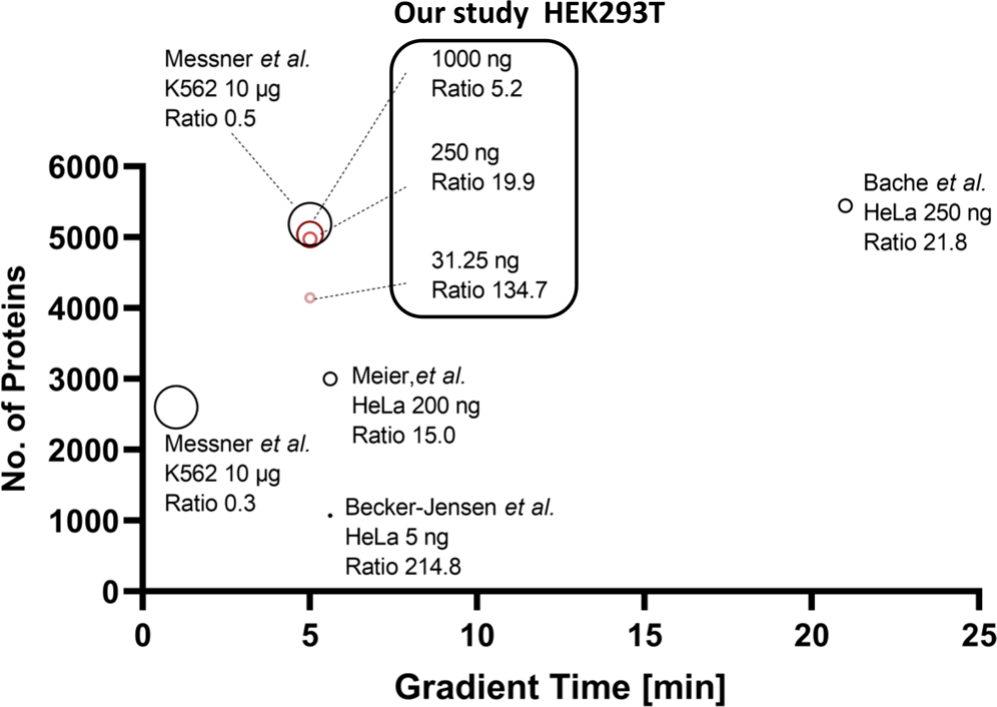
Comparison between our method and previous methods of protein identifications. The graph shows protein identifications for different gradient times in individual methods. Previous methods were referred from refs 9, 12, 13, and 14. The protein identification ratio was calculated from protein identifications divided by digest amount [ng]. Dot size indicates loaded digest amount.

We identified 5,186 proteins and quantified 4930 proteins in triplicate using a 1000 ng cell digest (Figure 2B). Of these, 3880 and 2467 proteins indicated CV < 0.2 and 0.1, respectively (median CV of 10.2%). The CV% of proteins was not significantly different from that of previous ultrafast methods (median CVs of 10.3 and 6.4%^12,13^). Among the identified proteins, the dynamic range of intensity was >10^4^ (Figure 2C, left panel). To investigate the proteomic coverage of our method, we focused on the number of identified minor component proteins, represented as kinases, transcriptional factors, and small GTPases. In total, 139, 186, and 99 for kinases, transcription factor-related proteins, and GTPases, respectively, were identified using our method (Figure 2C, right panel). Kinase and transcription factor identifications were fewer than those of our previous method (502 and 1029 identifications, respectively: 90 min gradient at a flow rate of 100 nL/min).^7^ Given the difference in measurement time, we considered that our method indicated an adequate depth of proteome coverage, especially in clinical applications. Our method is suitable for the routine measurement of numerous samples because of its analytical throughput and may be effective for the diagnosis and screening of biomarker candidates. As shown in Figure 3, our method showed good protein identification rates of 134.7, 19.9, and 5.2 proteins/ng for 31.25, 250, and 1000 ng digest injections, respectively. These results imply that our method requires a lower sample volume for protein extraction. Consequently, our method reduces sample volume, acquisition time, and data size.

### High-Throughput Proteomic Application by a Chemical Stimulation Assay of Cells

Drug screening should be performed for many samples; thus, HT methods are required. One of the important aims of applying proteomics to HT methods is to capture the differences in protein profiles as a consequence of stimulation. To obtain findings for future HT proteomic applications, we investigated whether our method enables continuous measurement of multiple samples and captures biological alterations in proteome profiles via a cell stimulation assay using eight chemical substances. Subsequently, we performed a pathway analysis for each stimulation. Information on the pathway analysis with statistical significance is summarized in Table S3. The adequacy of the assay was verified by observing the bioactivity of LPA(14:0) and S1P stimulation. LPA(14:0) and S1P are bioactive lysolipids, and their G-protein-coupled receptors (GPCRs) are ubiquitously expressed in human cells.^25−27^ LPA and S1P receptor (LPAR and S1PR) signaling regulate various biological functions, including cytoskeleton and adherens junction assembly, mediated by actin polymerization, followed by GTPase activation.^25,26^ In 0.1 μM LPA stimulation, upregulation of the “Regulation of actin cytoskeleton” pathway suggested that LPA stimulation activates LPAR/Rho/Rho-associated protein kinase signaling.^26^ In addition, PLCB3 (1-phosphatidylinositol 4,5-bisphosphate phosphodiesterase β-3; phospholipase C-β3) was included in upregulated pathways by LPA(14:0) stimulation (Table S3). LPAR is known to activate LPAR/phospholipase C-β3/AKT/ERK signaling.^26,28^ These observations support the idea that LPAR signaling is activated by LPA stimulation. Although annotated pathways such as S1PR signaling were not found in this assay, an increase in actin- and tubulin-related proteins, including ACTB (β-actin), FLNA (Filamin A), and TUBA1A (Tubulin Alpha 1a) (Table S3), was supported to enhance membrane trafficking and actin polymerization via SIPR signaling activation. Our findings are consistent with those of previous studies, which support the adequacy of our method. S1P stimulation resulted in the highest number of both up- and downregulated pathways: 8, 7, and 14 pathways at 0.1, 1.0, and 10 μM, respectively (total 29 pathways) (Table S3). These results suggest that S1P-S1PR signaling regulates numerous biological and physiological functions. For other stimulations, 8, 10, 7, 9, 11, and 8 pathways were annotated by Sph, PI3P, PI4P, PI5P, IP3, and IP4 stimulation, respectively (Table S3). Substance-specific altered pathways were observed in each stimulation such as “Protein export” for 10 μM PI3P and “Ubiquinone and other terpenoid-quinone biosynthesis” for 1.0 μM PI4P stimulation, whereas several altered pathways were overlapped in individual stimuli (Table S3). Thus, each stimulation may have a different effect on intracellular signaling. However, it is unclear how these substances act in the extracellular space as their GPCRs have not yet been identified. Thus, further investigation is required to clarify the mechanisms underlying the bioactivity.

**Figure 4:**
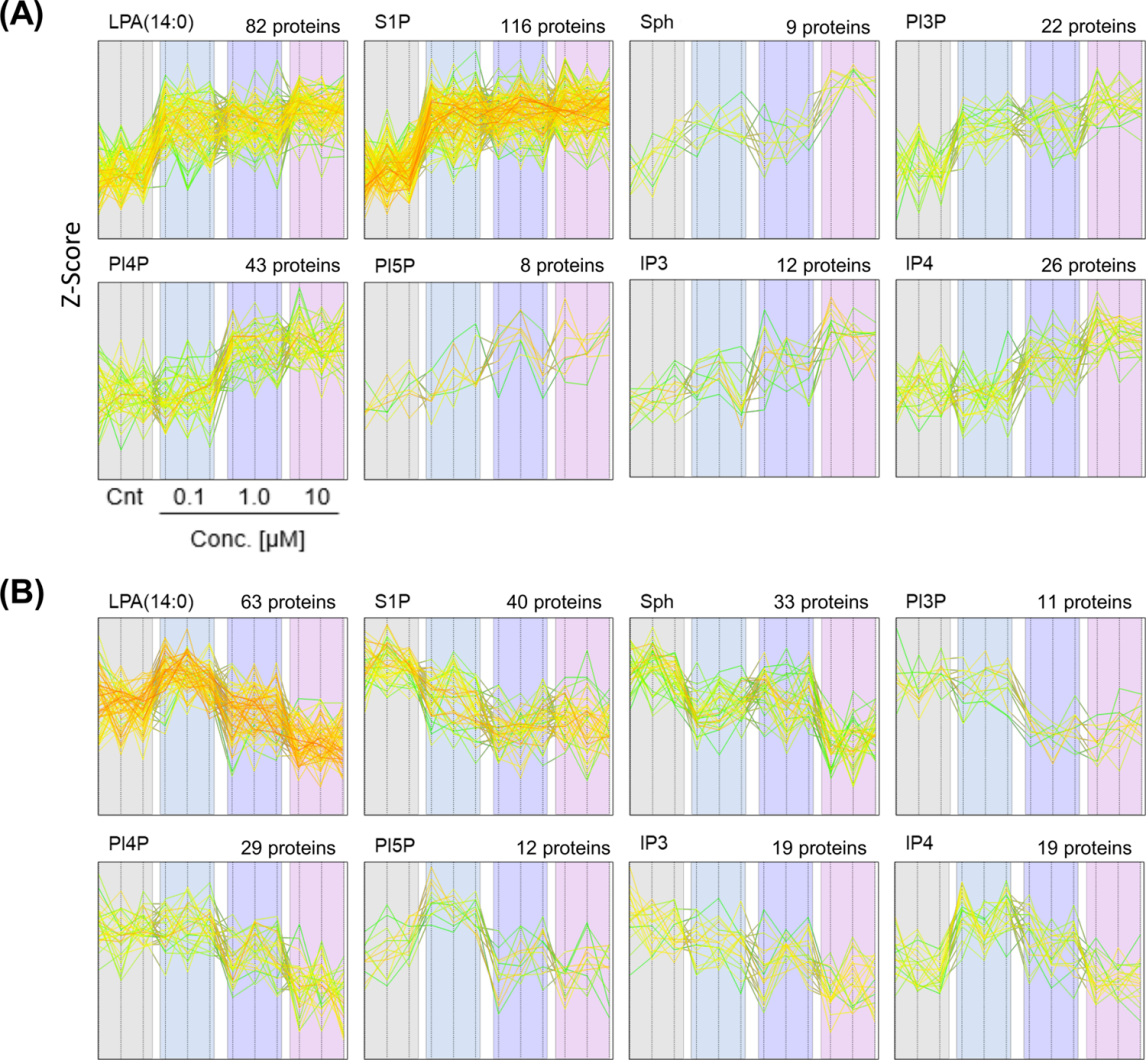
Dose-dependent responsive proteins by chemical stimulus in HEK293T cells. The profile plots indicate the z-score with dose-dependent manner increased (A) and decreased (B) proteins by stimulations of eight substances. The number of altered proteins is shown above each profile plot. The stimulation assay was performed in triplicate.

**Figure 5:**
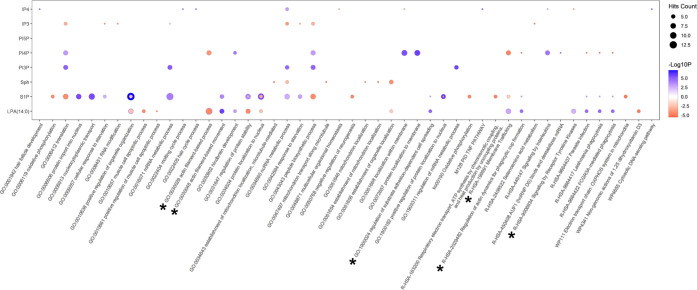
Representative dose-dependent altered GO terms in HEK293T cells by each substance stimulation. The dot plot indicates dose-dependent manner increased and decreased GO terms with top 5 *p*-values in each stimulation. Notably, top 5 GO terms in other stimulations are also displayed, regardless of the *p*-value rank. Hit count represents the number of overlapping counts among individual stimulations. Blue and red dots show dose-dependent manner increased and decreased GO terms, respectively.

Additionally, we performed hierarchical clustering and GO enrichment analysis of the proteins with statistical significance for each substance (Figure S3A–H). In this analysis, we found that a dose-dependent response was an efficient bioactivity for each substance. Thus, we selected two clusters that indicated that the bioactivity increased or decreased in a dose-dependent manner after each stimulation (Figure 4A,B). The number of significantly altered proteins is summarized in Table S4. Predictably, actin filament, membrane traffic, and cell adhesion-related terms (GO:0030048, GO:0030039, GO:1900024, R-HSA-2029482, R-HSA-199991, indicated by asterisks in Figure 5) were significant GO terms in both LPA(14:0) and S1P stimulation. In addition, “signaling by receptor tyrosine kinase (R-HSA-9006934)” was significantly increased after LPA(14:0) stimulation. LPA signaling induces the transactivation of an epidermal growth factor receptor (EGFR),^29,30^ which is a receptor tyrosine kinase. In this assay, EPS15, CRK, MID, GTF2F2, PTK2, and WASF2 were annotated with “signaling by receptor tyrosine kinase (R-HSA-9006934)”. LPA(14:0) stimulation significantly increased the levels of these proteins compared with those of the control (EPS15, PTK2, and CRK at 10 μM LPA(14:0); MDK at 0.1 and 10 μM LPA(14:0); GTF2F2 and WASF2 at 1.0 μM LPA(14:0)) (Figure S4). The EGFR levels were not significantly different between the LPA stimulation and control groups (Figure S4). We investigated the bioactivity of Sph, PI3P, PI4P, PI5P, IP3, and IP4 using our HT proteomic platform (Figure 5). For instance, Sph stimulation was found to be involved in mitochondrial localization (GO:0051654, GO:0051646, GO:0047497, and GO:0034643). IP3 stimulation was mainly associated with translation and peptide synthesis (GO:0043043 and 0006412). Our results may be helpful for researchers planning to perform HT screening using these compounds. Consequently, our method enabled the measurement of approximately 80 samples and further alteration of proteomic profiles by chemical stimulation.

## Conclusions

In this study, we revealed that the optimized DIA parameters are efficient for improving protein coverage and they enable us to capture the alteration of proteome profiles when using 5-min gradient LC and Q-Orbitrap MS. Of note, these DIA parameters show a high number of protein identifications even when a smallamount of digest is injected. To our knowledge, this study is the first to evaluate the detailed DIA parameters for HT proteomics using Q-Orbitrap MS. Overall, our results may provide helpful information for enhancing HT proteomic applications using Q-Orbitrap MS in future medical and medicinal fields.

## Supporting Information Available

Representative total ion chromatogram of 5-min gradient LC-MS/MS (Figure S1); re-analysis of data obtained from selected nine methods by analyzing Scaffold DIA (Figure S2); hierarchical clustering and GO enrichment analysis of HEK293T cell proteins stimulated by (A) LPA(14:0), (B) S1P, (C) Sph, (D) PI3P, (E) PI4P, (F) PI5P, (G) IP3, and (IP4) (Figure S3); levels of EGFR and proteins annotated with “signaling by receptor tyrosine kinase (R-HSA-9006934)” by LPA(14:0) stimulation (Figure S4) (PDF)
Number of identified proteins and peptides in triplicate runs for selected nine DIA methods by analyzing DIA-NN (Table S1); number of identified proteins and peptides in triplicate runs for selected nine DIA methods by analyzing Scaffold DIA (Table S2); information of annotated pathway with statistical significance for each stimulation (Table S3); summary of cell stimulation assay using eight chemical substances (Table S4) (XLSX)

## Notes

Mass spectrometry proteomics data were deposited in the ProteomeXchange Consortium via the jPOST partner repository^31^ with the dataset identifier PXD034227 for ProteomeXchange and JPST001513 for jPOST.
