# Supplementary material for: Optimization of Ultrafast Proteomics Using an LC-Quadrupole-Orbitrap Mass Spectrometer with Data-Independent Acquisition
Source: J Proteome Res. 2022 Aug 1;21(9):2085–93. doi: 10.1021/acs.jproteome.2c00121 (PMC9442788; doi:10.1021/acs.jproteome.2c00121)

Supporting Information

Optimization of Ultra-Fast Proteomics using an LC-Quadrupole-Orbitrap Mass Spectrometer with Data-Independent Acquisition

Masaki Ishikawa[1,†], Ryo Konno[1,†], Daisuke Nakajima[1], Mari Gotoh[2], Keiko Fukasawa[2], Hironori Sato[1], Ren Nakamura[1], Osamu Ohara[1], Yusuke Kawashima[1,*]

[1]Laboratory of Clinical Omics Research, Department of applied genomics, Kazusa DNA Research Institute, Kisarazu, Chiba 292-0818, Japan.

[2]Institute for Human Life Innovation, Ochanomizu University, Bunkyo-ku, Tokyo 112-8610, Japan.

[†]These authors contributed equally to this study.

Corresponding Author

*Yusuke Kawashima, Ph.D.
Laboratory of Clinical Omics Research, Department of Applied Genomics, Kazusa DNA Research Institute, Japan.

Address: 2-5-23 Kazusa-kamatari, Kisarazu, Chiba 292-0818, Japan

E-mail: ykawashi@kazusa.or.jp

Contents

Figure S1. Representative total ion chromatogram of 5-min gradient LC-MS/MS.

Figure S2. The re-analysis of data obtained from selected nine method by analyzing Scaffold DIA.

Figure S3. Hierarchical clustering and GO enrichment analysis of HEK293T cell proteins stimulated by (A) LPA, (B) S1P, (C) Sph, (D) PI3P, (E) PI4P, (F) PI5P, (G) IP3, and (IP4).

Figure S4. The levels of EGFR and proteins annotated with “signaling by receptor tyrosine kinase (R-HSA-9006934)” by LPA(14:0) stimulation.

Table S1. The number of identified proteins and peptides in triplicate runs for selected nine DIA methods by analyzing DIA-NN. (.xlsx)

Table S2. The number of identified proteins and peptides in triplicate runs for selected nine DIA methods by analyzing Scaffold DIA. (.xlsx)

Table S3. The information of annotated pathway with statistical significance for each stimulation. (.xlsx)

Table S4. Summary of cell stimulation assay using eight chemical substances. (.xlsx)

Figure S1. Representative total ion chromatogram of 5-min gradient LC-MS/MS.

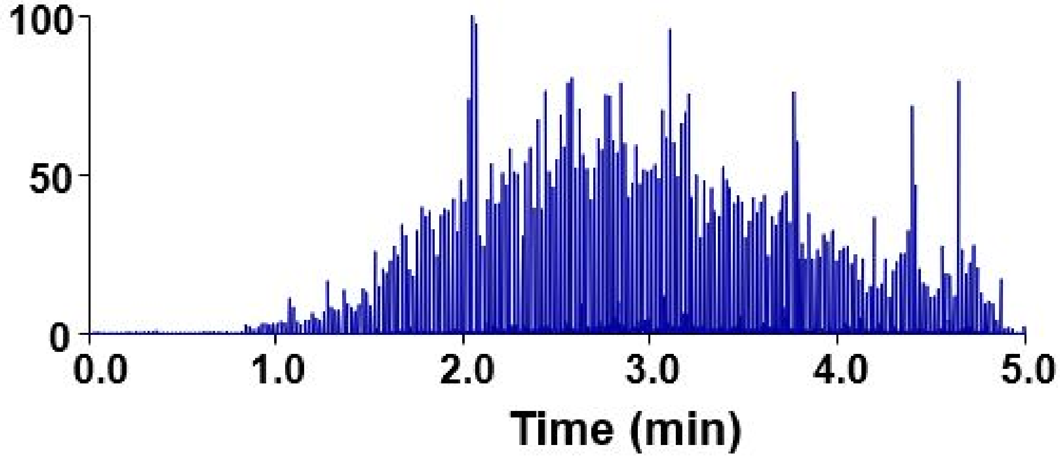

Figure S2. The re-analysis of data obtained from selected nine methods by analyzing Scaffold DIA. The bar graphs indicate the number of identified (A) proteins and (B) peptides using 500 ng cell digest with triplicate runs. Nine methods are comprising with narrow precursor mass ranges (120 Da: Res15k/W4/650–770, Res30k/W8/650–770, Res30k/W8/600–720, Res30k/W8/550–670, and Res30k/W8/500–620) and wide precursor mass ranges (240 Da; Res15k/W8/650–890, Res15k/W8/600–840, Res15k/W8/550–790, and Res15k/W8/500–740).

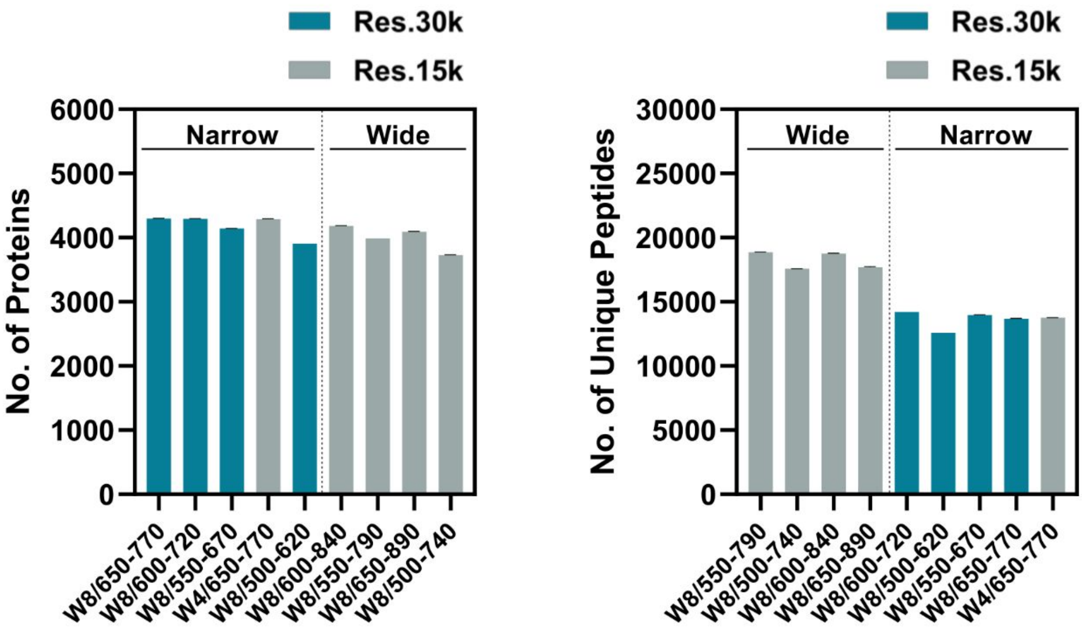

Figure S3. Hierarchical clustering and GO enrichment analysis of HEK293T cell proteins stimulated by (A) LPA(14:0), (B) S1P, (C) Sph, (D) PI3P, (E) PI4P, (F) PI5P, (G) IP3, and (IP4).

(A) LPA(14:0)

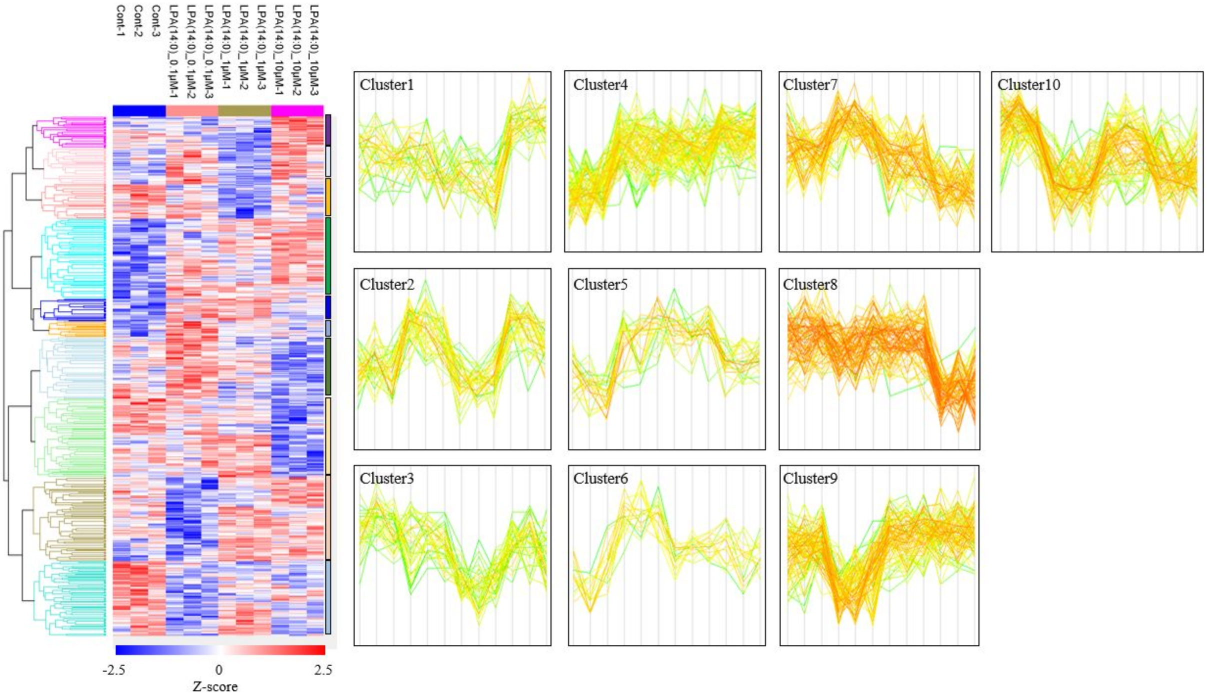

(B) S1P

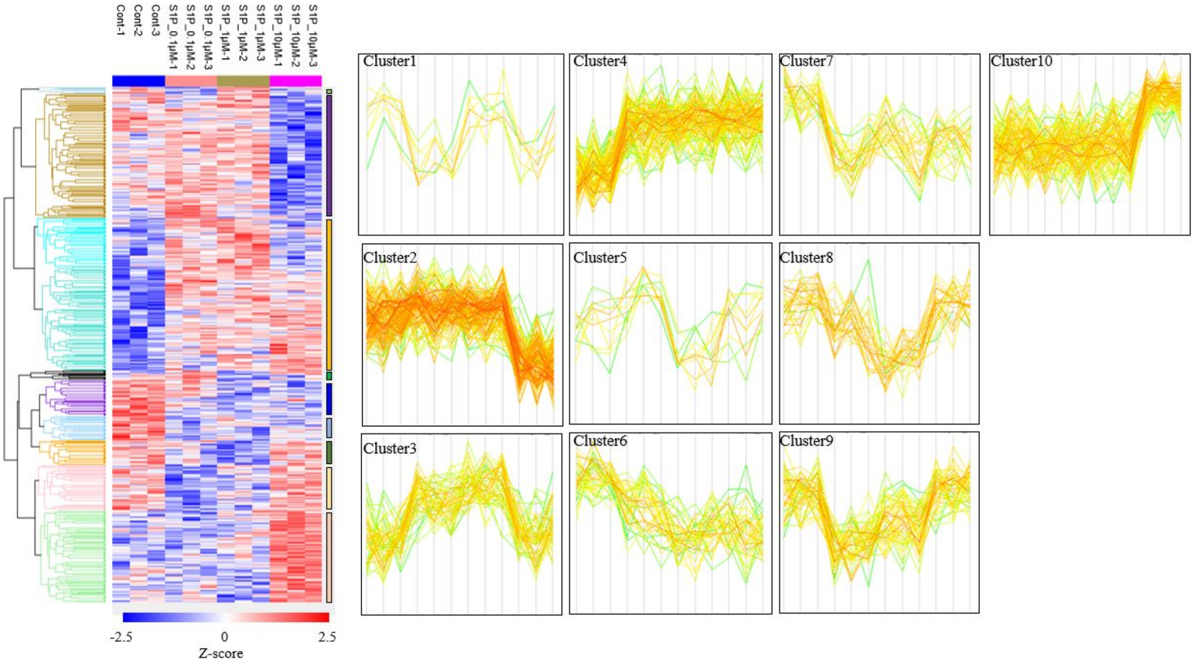

(C) Sph

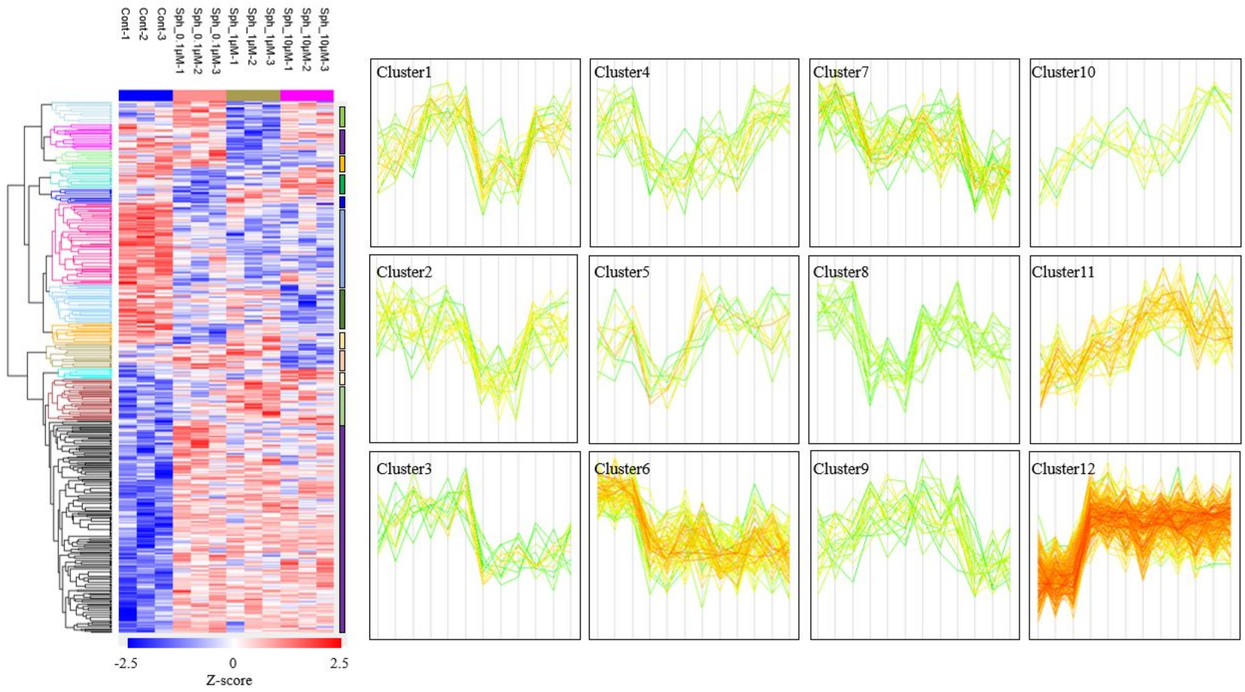
Cont-1
Cont-2
Cont-3
Sph_0.1μM-1
Sph_0.1μM-2
Sph_0.1μM-3
Sph_1μM-1
Sph_1μM-2
Sph_1μM-3
Sph_10μM-1
Sph_10μM-2
Sph_10μM-3
-2.5
0
2.5
Z-score
Cluster1
Cluster4
Cluster7
Cluster10
Cluster2
Cluster5
Cluster8
Cluster11
Cluster3
Cluster6
Cluster9
Cluster12

(D) PI3P

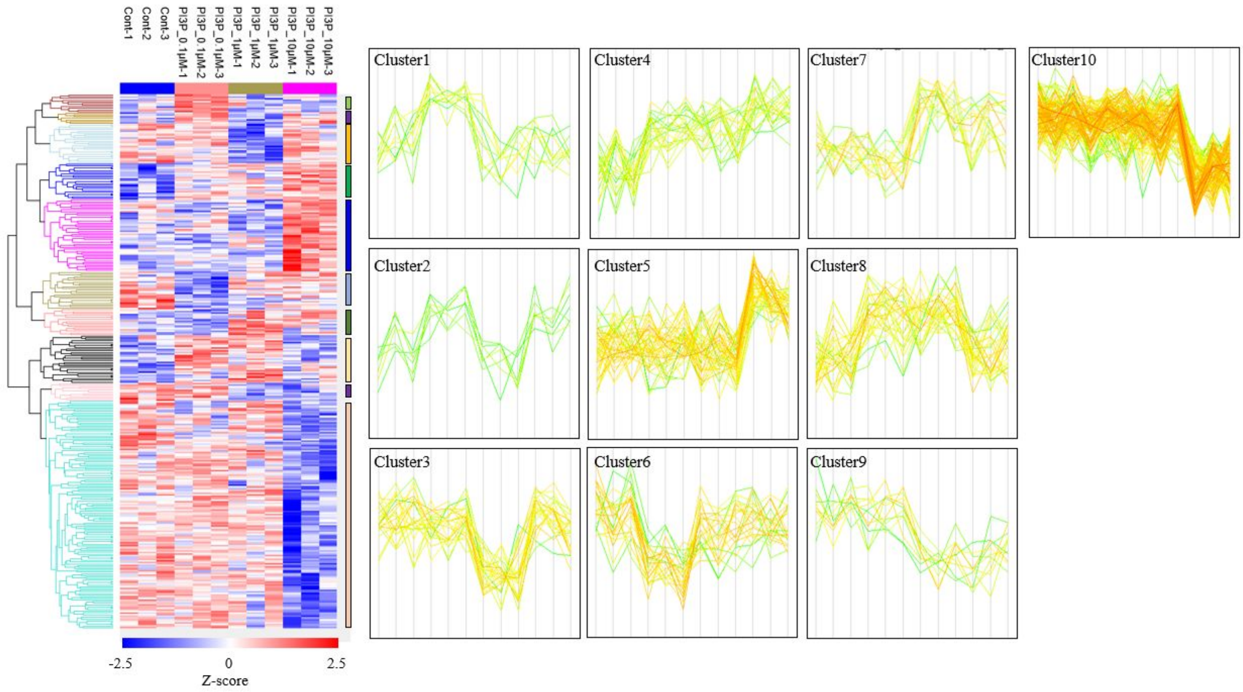
Cont-1
Cont-2
Cont-3
PI3P_0.1μM-1
PI3P_0.1μM-2
PI3P_0.1μM-3
PI3P_1μM-1
PI3P_1μM-2
PI3P_1μM-3
PI3P_10μM-1
PI3P_10μM-2
PI3P_10μM-3
-2.5
0
2.5
Z-score
Cluster1
Cluster4
Cluster7
Cluster10
Cluster2
Cluster5
Cluster8
Cluster3
Cluster6
Cluster9

Figure. S3 continue

(E) PI4P

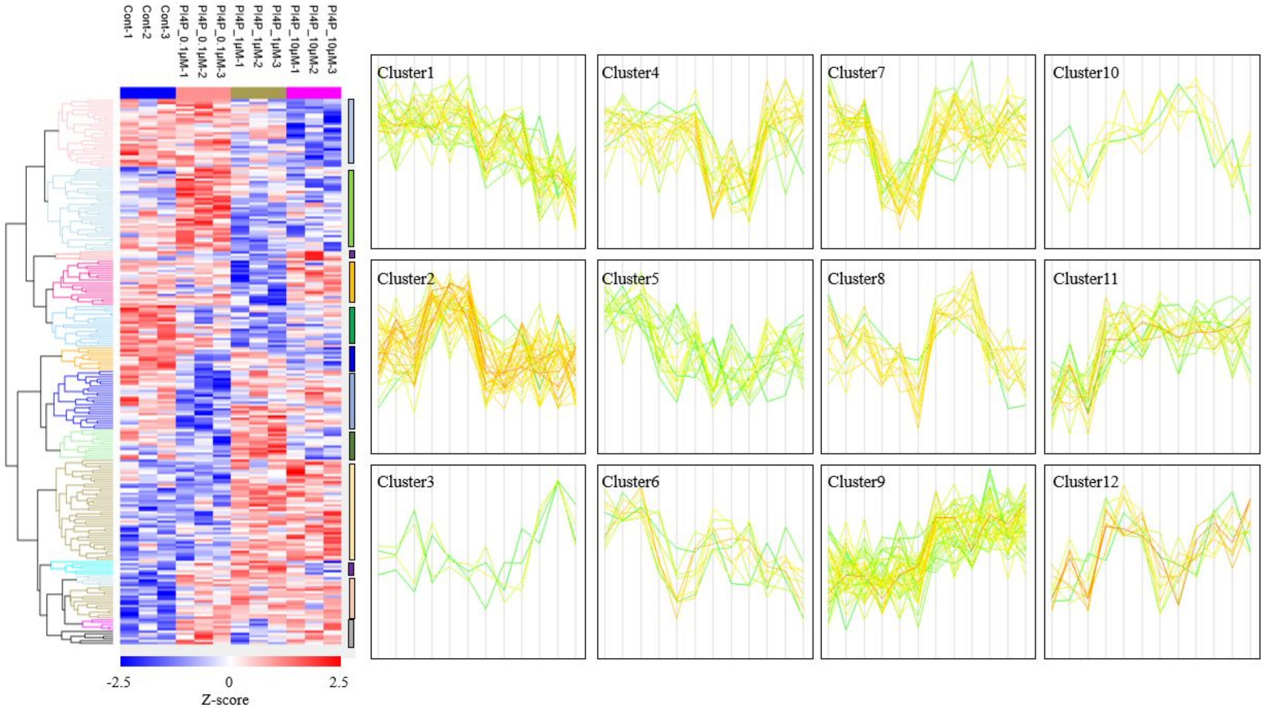

(F) PI5P

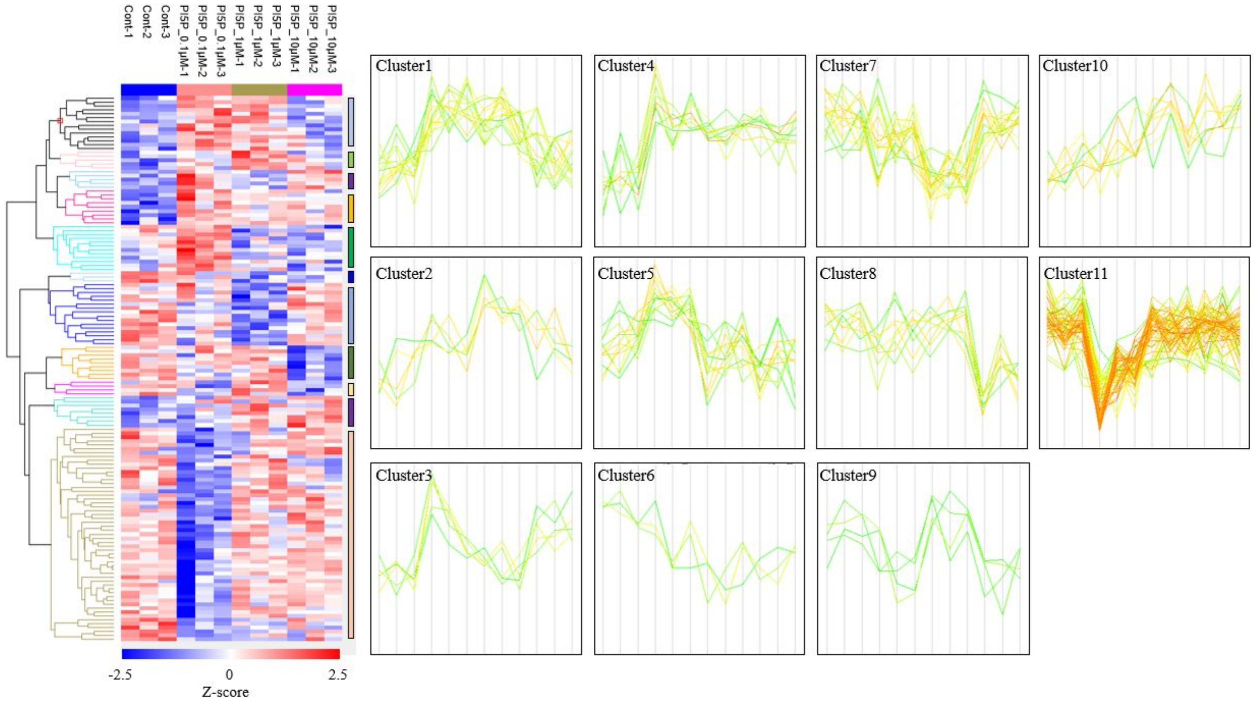

Figure. S3 continue

(G) IP3

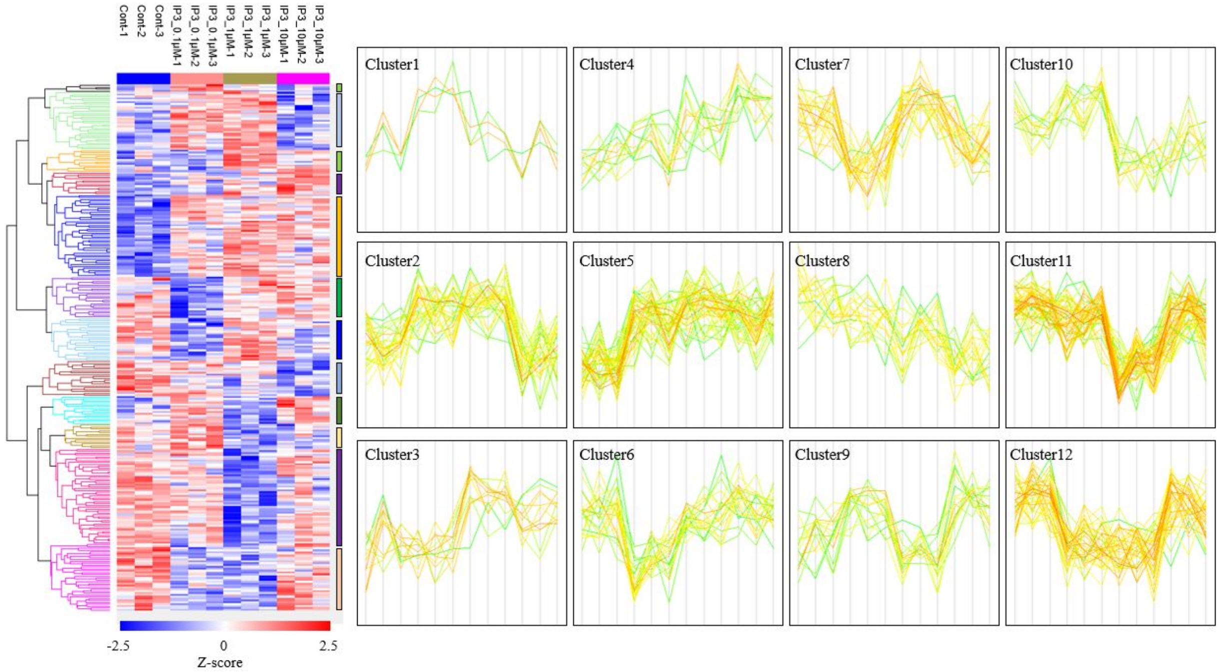

(H) IP4

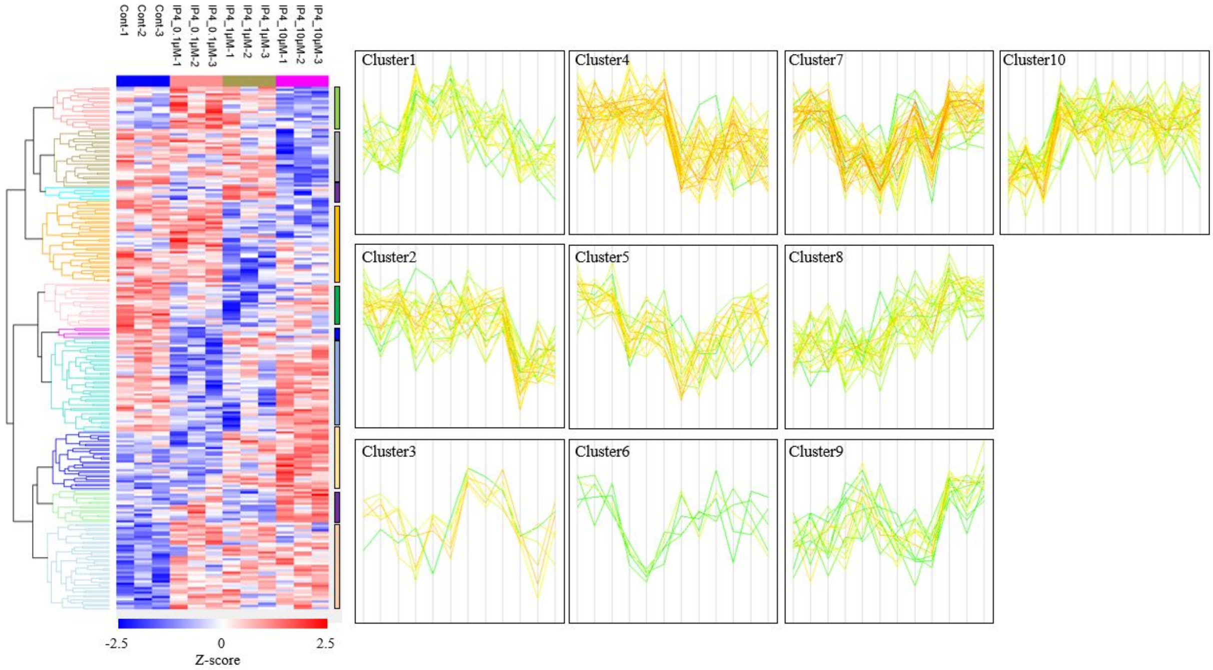

Figure S4. The levels of EGFR and proteins annotated with "signaling by receptor tyrosine kinase (R-HSA-9006934)" by LPA(14:0) stimulation. Statistical analysis was performed using one-way ANOVA followed by post-hoc Turkey's multiple comparison test (*$p < 0.05$; **$p < 0.01$).

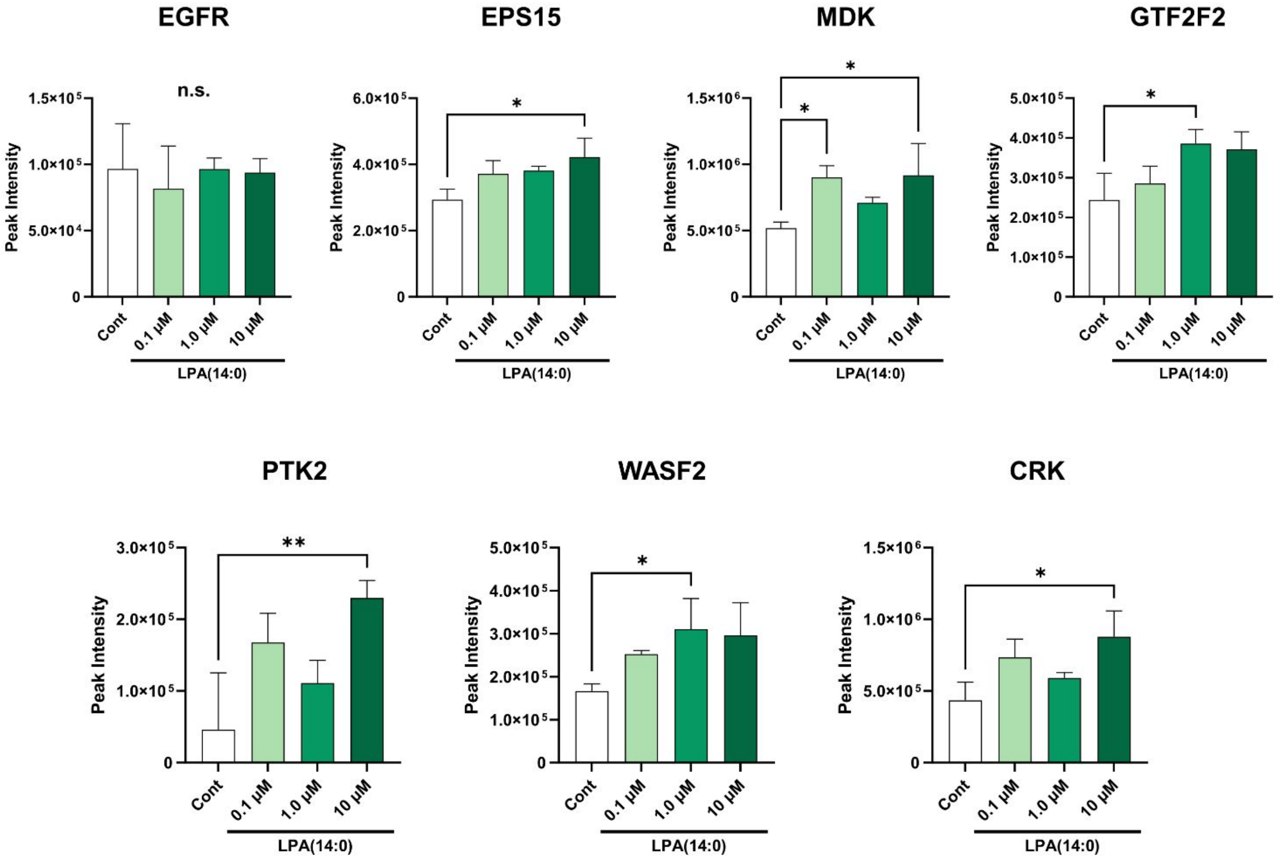

Supplement: Supplementary file 1 — pr2c00121_si_001.pdf [file pr2c00121_si_001.pdf]
